# Variable Number of Tandem Repeats (VNTR) analysis of *Flavobacterium psychrophilum* from salmonids in Chile and Norway

**DOI:** 10.1186/s12917-015-0469-7

**Published:** 2015-07-14

**Authors:** Patricia Apablaza, Øyvind J. Brevik, Svein Mjøs, Samuel Valdebenito, Pedro Ilardi, Juan Battaglia, Inger Dalsgaard, Are Nylund

**Affiliations:** Fish Diseases Research Group, Department of Biology, University of Bergen, P.O. 7803, N-5020 Bergen, Norway; Nofima, Kjerreidviken 16, 5141 Fyllingsdalen, Bergen Norway; Veterquímica, Camino Melipilla 5641, Cerrillos, Santiago Chile; National Veterinary Institute, Technical University of Denmark, Bülowsvej 27, 1870 Frederiksberg C, Denmark

**Keywords:** *Flavobacterium psychrophilum*, Characterization, VNTR, Genotyping, PCA, Salmonid, Wild, Farmed

## Abstract

**Background:**

*Flavobacterium psychrophilum* causes serious fish diseases such RTFS and BCWD, affecting the aquaculture industry worldwide. Commercial vaccines are not available and control of the disease depends on the use of antibiotics. Reliable methods for detection and identification of different isolates of this bacterium could play an important role in the development of good management strategies. The aim of this study was to identify genetic markers for discrimination between isolates. A selection of eight VNTRs from 53 *F. psychrophilum* isolates from Norway, Chile, Denmark and Scotland were analyzed. The results were compared with previous work on the same pathogen using MLST for genetic differentiation.

**Results:**

The VNTR analysis gave a separation between the *F. psychrophilum* isolates supporting the results of previous MLST work. A higher diversity was found among the Chilean isolates compared to those from Norway, which suggests a more homogenous reservoir in Norway. Transgenerational transmission of *F. psychrophilum* from other countries, exporting salmon embryos to Chile, may explain the differences in diversity. The same transmission mechanisms could also explain the wide geographical distribution of identical isolates in Norway. But, this could also be a result of movement of smolts and embryos. The selected VNTRs are stable genetic markers and no variation was observed after several passages on agar plates at different temperatures.

**Conclusions:**

These VNTRs are important additions for genotyping of *F. psychrophilum* isolates. Future studies on VNTRs of *F. psychrophilum* should include isolates from more host species from a wider geographical area. To get a more robust genotyping the VNTRs should be used in concert with MLST. Future studies of isolates with high and low virulence should focus on identifying virulence markers using VTNRs and MLST.

**Electronic supplementary material:**

The online version of this article (doi:10.1186/s12917-015-0469-7) contains supplementary material, which is available to authorized users.

## Background

*Flavobacterium psychrophilum* causes bacterial cold water disease (BCWD) and rainbow trout fry syndrome (RTFS) in the fresh water phase of salmonid production worldwide [1]. Although some commercial vaccines are available (J. Battaglia com. pers.) flavobacteriosis is still a major problem in the salmonid production in Chile, and the control of the disease is dependent on the use of antibiotics [2]. Another approach is the use bacteriophages that have been tested with promising effects and use of probiotics and modified fish diets represent other options [3, 4].

Since the first reports of flavobacteriosis, the disease caused by *F. psychrophilum* (former name *Cytophaga psychrophila*) in the 1940s in North America, the pathogen has emerged in several countries causing severe outbreaks on salmonids and other fresh water species [5, 6]. It has been shown that *F. psychrophilum* can be transgenerational transmitted and, to prevent spreading and disease outbreaks it is necessary to develop specific and sensitive methods for detection of the bacterium [7].

Differences in virulence among *F. psychrophilum* isolates will have implications for management strategies and vaccine development [8]. These management strategies are dependent on the correct identification of the virulence and the source of origin the isolates. Based on existing knowledge the best approach for developing such identification tools will to involve knowledge about the genetics of the bacteria. Several methods have been developed for genetic differentiation of other fish pathogenic bacteria [9, 10]. The most commonly used methods are restriction fragment length polymorphism (RFLP), random amplified polymorphism (RAPD), 16S rDNA sequencing, housekeeping genes sequencing, MLST and VNTRs [11–17]. These methods or a selection of them can also be used for identification of host specificity and studies of geographical distribution, molecular epizootiology, and antibiotic resistance.

Use of variable number of tandem repeats (VNTR) are among the most discriminating and least time consuming of the genotyping methods. This method has been widely used during the last 15 years for genotyping of bacteria causing diseases in humans, domestic animals and vegetables [18–22]. In aquaculture, the method has been used for genotyping of Atlantic salmon *Salmo salar* [23], and the parasitic copepod *Caligus rogercresseyi* Boxshall and Bravo 2000 [24], but also for genotyping of other fish pathogenic bacteria [14, 25–27].

The aim for this study was to identify VNTRs that can be used to generate a genotyping system for separation of *F. psychrophilum* isolates. This VNTR system was applied to a set of isolates from Norway and Chile.

## Results

### Variable number of tandem repeats (VNTR)

The 12 pairs of VNTR-primers were tested using DNA extracted from the type strain (NCIMB 1947), five Norwegian, and five Chilean isolates of *F. psychrophilum*. Among this initial group of 11 isolates, only eight of the 12 amplified VNTRs loci showed variation. The remaining four (VNTR-2, VNTR-3, VNTR-4 and VNTR-12), were not tested for the other isolates. For some isolates, the primers for VNTR-7 and VNTR-13 did not produce the expected PCR products, but a new set of primers yielded these products with the exception of the isolate No10-35-T-W. The accession numbers of all the obtained VNTRs are included in the Additional file 1.

The eight VNTR loci gave variation from one to nine repetitions among the isolates (*n* = 53) included a table with all the allelic VNTR profiles for all the isolates is presented in the Additional file 2. The type strain and some isolates from Norway and Chile present two types of deletions in VNTR-7, where the complete repetitive sequence is TTAAAAA. One variant is TAAAA and the other is AAAA with no repetitions. The VNTRs, related to the genome of JIP02 /86, show that they are located in both intergenic and intragenic positions. In the case of VNTR-10, the repeats are located in the gene associated with gliding motility and encode for the GldL protein (Table 3). No variation in the number of repeats and nucleotide positions in the eight VNTRs loci studied were observed when the VNTR stability of isolate No12-49-As-Op was tested after 12 passages on agar plates at two different temperatures (4 °C and 15 °C).

**Table 1:** New isolates of *F. psychrophilum* included in this study in addition to the published in Apablaza et al. 2013

Code	Country	County	Year	Water source	Host	Wild/farmed	Tissue
No11-46-As-Sp	Norway	Hordaland	2011	fresh water	Atlantic salmon	wild	spleen
No12-49-As-Op	Norway	Nordland	2012	fresh water	Atlantic salmon	farmed	operculum
No12-50-As-Mo	Norway	Møre og Romsdal	2012	fresh water	Atlantic salmon	farmed	mouth
No12-51-As-W	Norway	Møre og Romsdal	2012	fresh water	Atlantic salmon	farmed	wound
Sc10-47-As-K	Scotland	NA	2010	fresh water/loch	Atlantic salmon	farmed	kidney
Sc11-48-Rt-Sp	Scotland	NA	2011	fresh water/ burn	rainbow trout	farmed	spleen
Dn94-52-Rt-Sp	Denmark	Western Jutland	1994	fresh water	rainbow trout	farmed	spleen
Dn08-53-Rt-K	Denmark	Himmerland	2008	fresh water	rainbow trout	farmed	kidney

Isolates of *F. psychrophilum* from Norway, Scotland and Denmark, collected between 1994 to 2012, from different water sources, fish species, farmed or wild fish and tissues. Codes indicate the country of isolation, *No* Norway, *Sc* Scotland, and *Dn* Denmark; year (1994 to 2012); number of isolate (46 to 53); fish species, A. *As* salmon, *Rt* rainbow trout, and tissue, *Sp* spleen, *Op* operculum, *Mo* mouth, *W* wound, *K* kidney. *NA* no information is available

**Table 2:** Primers used to performed the amplification of the VNTRs regions of *F. psychrophilum* included in this study

Primer name	Sequence (5’. > 3')	Tm°C	Target	Amplicon size (bp)
FpVNTR1/F	GATGGCACGAATGTCGTCGC	57	VNTR no 1	428
FpVNTR1/R	TGGTTTCGGAGCATCGCCTC	57		
FpVNTR2/F	CATGGCAACACCTTGCGCTC	57	VNTR no 2	631
FpVNTR2/R	TCCCCAATACCGTTTGGCGT	56		
FpVNTR3/F	GGCCTGGGCTATTCGTTGCA	57	VNTR no 3	320
FpVNTR3/R	CCCATTGCCGAAACTACGAGC	56		
FpVNTR4/F	GCCATCGGGGAAAACAGAGC	57	VNTR no 4	412
FpVNTR4/R	TGCAACCGTACCAACAGGCA	56		
FpVNTR5/F	CACAACCGAATTGCACGCCA	57	VNTR no 5	546
FpVNTR5/R	TCGGCTGGACAAGCGCTTTT	57		
FpVNTR6/F	TAACGCCGCTGCTTGTGCTA	57	VNTR no 6	685
FpVNTR6/R	TGGCTTCTTACGCTGGTATGCA	56		
FpVNTR7/F	TGCAACGCAGATGACACGGA	57	VNTR no 7	580
FpVNTR7/R	AGGAGTAAGGCTTCTTCCCCGT	57		
FpVNTR8/F	TGCTCTTTCGCCATAGCGGT	56	VNTR no 8	737
FpVNTR8/R	CCAACGGCAAATGCTCCCAT	56		
FpVNTR9/F	CCCGTAAATAAAACTCACAGACAG	52	VNTR no 9	670
FpVNTR9/R	TGGTGCTATCCCGCCAAATG	55		
FpVNTR10/F	ACTCCATTTGAGCAGCTGCC	55	VNTR no 10	417
FpVNTR10/R	TTCGCTTACGGTATGGGAGCG	57		
FpVNTR12/F	GCAAACGGCAATGGCCACAT	57	VNTR no 12	445
FpVNTR12/R	GCTCGCGTTCCTTTCTCGGT	57		
FpVNTR13/F	GCCCAATGGCTCAAATCGTCG	57	VNTR no 13	378
FpVNTR13/R	CGCATGACCTCATCTCGGGT	56		
FpVNTR7_2/F	TGCCAATGCGGGTGAAAAG	57	VNTR no 7	1140
FpVNTR7_2/R	TCGGCACCCAATTGCAATCCT	57		
FpVNTR13_2/F	CCAACTTGGGTTCAGGTTGTGAA	58	VNTR no 13	895
FpVNTR13_2/R	ACACCAAACTACCCCGAAATGGA	58		

The last to set of primers were used for a few isolates analysis

**Table 3:** Location of the Variable Number of Tandem Repeats in the genome of *F. psychrophilum* JIP02/86

VNTR	Repeat sequence	Location
FpVNTR1	AATAGGC	Intergenic
FpVNTR2	AAAGTTT	Intergenic
FpVNTR3	AAATCTA	Intergenic
FpVNTR4	AATTCAT	Intergenic
FpVNTR5	TTAGGCA	Intergenic
FpVNTR6	TTTCTAAT	Intergenic
FpVNTR7	TTAAAAA	Intergenic
FpVNTR8	AATCTGA	Intergenic
FpVNTR9	GTTT	Intergenic
FpVNTR10	CTTTTACTT	Intragenic/gene for gliding motility protein GldL
FpVNTR12	ATTTTAG	Intergenic and intragenic
FpVNTR13	AATCCACA	Intergenic and intragenic

The obtained sequencing data for the VNTRs were used to prepare profiles for the isolates of *F. psychrophilum*. These profiles revealed 25 different VNTR sets (VS) (Supplementary material). This VNTR polymorphism constituted the basis for the establishment of a multiple-loci VNTR analysis (MLVA) system for separation of the isolates.

### Grouping of the *F. psychrophilum* isolates

**Fig. 1:**
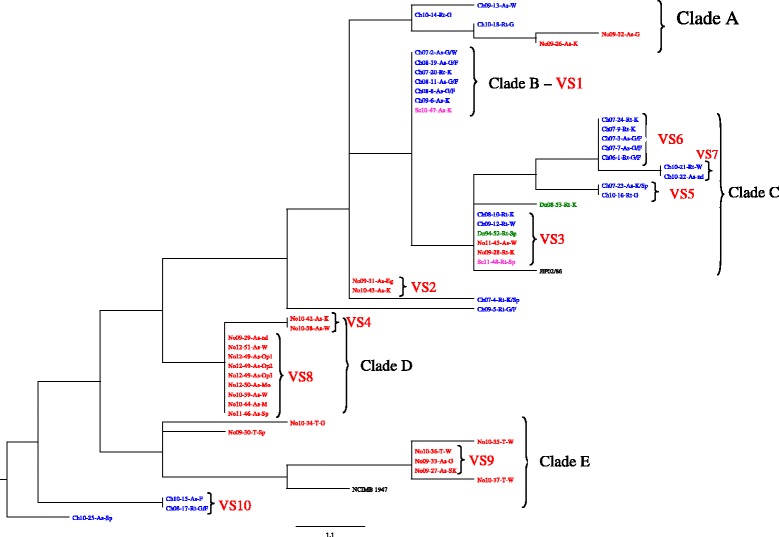
The genetic relationships of the isolates included in this study. Relationship among 53 isolates of *F. psychrophilum* based on allelic differences at eight VNTR loci. This unrooted ultrameric NJ tree show know topography between Norwegian and Chilean isolates. Codes of are explained in the table included in Additional file 3

**Fig. 2:**
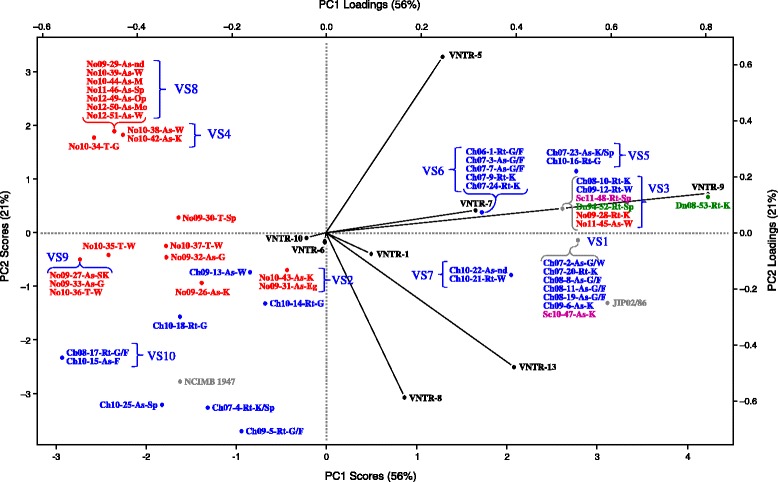
PCA plot of all 53 isolates of *F. psychrophilum,* from Norway (red), Chile (blue), Denmark (green) and Scotland (violet), type strain NCIMB 1947 and of *F. psychrophilum* JIP02/86. The figure also includes the different VNTRs analysed. The isolate No12-49-As-Op tested for VNTR stability (4 and 15 °C) showed the same VT profiles in both temperatures Additional file 2, therefore they are not shown in this figure

The relationships between the *F. psychrophilum* isolates, based on VNTR analysis, are presented in Fig. 1. The majority of the isolates from Norway and Chile group in separated clades. However, eleven European isolates are found among the Chilean clades (A, B, and C), while the majority of the Norwegian isolates group in distinct clades with the type species. A second group of the Chilean isolates constitute the most basal clade (F). These results are also to a large extent supported by the PCA, Fig. 2.

Among the 53 isolates included in the study only one group (clade D) were obtained from one host species (*Salmo salar*) only. Another clade (C) was dominated by isolates from rainbow trout and a third clade (B) by isolates from Atlantic salmon. Both these clades consisted of a majority of Chilean isolates. Clade E consists of isolates obtained from wild trout (*Salmo trutta*) and Atlantic salmon from river Eira, western of Norway, and the type species (NCIMB 1947) from coho salmon. The PCA confirm this distribution.

Isolates of *F. psychrophilum* were obtained from both healthy fish and fish showing signs of flavobacteriosis. Based on VNTR analysis none of the clades show clear tissue specificity. However, both analyses identified one group of possible pathogenic Norwegian isolates obtained from Atlantic salmon suffering flavobacteriosis (clade D, VS8, in Figs. 1 and 2). Possible pathogenic isolates from moribund fish collected in four different countries are also present in clade C. Clade E consists of isolates from both healthy and moribund wild salmonids.

## Discussion

Several techniques, based on nucleotide sequencing targeting different genes, have been employed in attempts to separate isolates of *F. psychrophilum,* and with highly variable results [28–30]. Among these, multi locus sequence typing (MLST) has been among the most promising methods [31, 32]. Results from these studies have demonstrated the existence of clonal complexes related to host species [32, 33], while no host species correlation was found in isolates from rainbow trout in Swiss farms [34].

MLVA based on VNTRs is another method that has showed promising results as an approach for separation of strains of other fish pathogenic bacteria [35]. In the search for genetic markers that may help to understand the epidemiology of *F. psychrophilum*, such a MLVA system based on eight VNTRs was established. A total of 53 isolates from different geographical locations, but with the majority obtained in Norway and Chile were used in the analysis. This is the first VNTRs based genotyping system for *F. psychrophilum*.

The present study shows the same separation between *F. psychrophilum* isolates from Norway and Chile, as that documented in the previous MLST work [36]. The VNTR method used confirmed also the high diversity of the Chilean isolates shown by MLST, and other work by Avendaño et al. [37] including a large number of isolates. In contrast, the Norwegian strains had low variability, which was also observed in another MLST study based on a large collection of isolates from Nordic countries [28]. The larger variation among *F. psychrophilum* isolates from Chile compared to the more limited variation seen among Norwegian isolates suggest that the salmon industry in the two countries are exposed to different reservoirs of the bacterium. A major difference between the two countries is that Norway has natural populations of Atlantic salmon and trout, while no salmonids are naturally occurring in Chile, but have been imported from several different countries and continents over several years [38]. The larger variation observed in Chile could be due to transgenerational transmission of *F. psychrophilum* from the different countries producing embryos from salmonids that are exported to Chile. The Chilean production of salmon eggs, during the expansion of the aquaculture industry, was not enough to meet the requirements of the industry, and a massive import of salmon embryos from North America and Europe continued for many years until the ISA outbreaks in 2007 [38]. This international commerce of embryos has resulted in the import of fish pathogens, such as ISA virus, IPN virus and piscine reovirus, PRV, to Chile [39–41]. Avendaño et al. [37] using MLST on 91 *F. psychrophilum* Chilean isolates suggested also that this bacterium was introduced from Europe and North America to Chile.

Studies of other bacterial pathogens using both MLST and VNTR typing systems, have been giving contradictory results. In a study on *Chlamydia felis* including isolates from different geographical origins, the discriminatory power of the MLVA scheme was superior to MLST [42]. However, a study of the human pathogen *Vibrio vulnificus*, gave the same separation for both MLST and VNTRs typing systems [43] which shows that the results obtained for *F. psychrophilum* are not unique. There are few reports using VNTRs to explain the geographical distribution of bacterial animal pathogens [44] and even less such studies on fish. The MLVA typing system that was applied to discriminate isolates of the Atlantic cod pathogen *Francisella noatunensis* subsp. *noatunensis*, gave a much better resolution compared to a study of housekeeping genes [26, 45]. A later study arrived at the same conclusion when using MLVA compared to PFGE method on a larger data set including 91 isolates of the same bacterium [46].

In the present study the resolution given by the VNTRs could not be used to discriminate among the strains from different fish hosts species with the exception of a cluster of *F. psychrophilum* collected from Atlantic salmon (clade D, VS4 and VS8). This cluster is formed by several farmed and wild strains from the North to the South of Norway and the pattern could be a result of movements of infected Atlantic salmon embryos and smolts during industrial production. Traditionally all brood fish production sites and the major production of smolts are located in western Norway, which mean that large quantities of salmon embryos and smolts are moved north. Hence, to a certain degree, the same transmission mechanisms as the long distance transmission from the northern to the southern hemisphere could explain the pattern of transmission in Norway. The extensive transmission of *F. psychrophilum* in connection with farming of salmonids could have blurred a previous existence of host specific strains of this bacterium, ie. a high infection pressure in farming areas could possibly explain presence of Atlantic salmon specific strains in rainbow trout and vice versa. However, the existence of a clonal complex connected to rainbow trout has been suggested to exist in Norway [28]. The existence of host specific strains of other fish bacteria, as *Yersinia ruckeri*, has also been shown using MLST [47], while no host specificity were observed in a study of 41 isolates from *Renibacterium salmoninarum* from Atlantic salmon and rainbow trout [27]. Future studies, including a larger number of *F. psychrophilum* isolates analysed by VNTRs may, however, give a better understanding of the genetic relationships between fish hosts and *F. psychrophilum.*

There are few reports in the literature concerning the stability of VNTR in bacteria subcultures at different temperatures. A report on *F. noatunensis* ssp. *noatunensis*, showed stability on the VNTRs selected loci over 3 years [46], although a previous work on the same pathogen reports instability in one VNTR after passages at different temperatures [26]. Another study of VNTR from *C. felis* showed high stability after 10 passages [42]. In the present study no variation was observed in a *F. psychrophilum* isolate after 12 passages at 4 and 15 °C, suggesting that the selected VNTRs could be considered as stable genetic markers.

The present study was not able to separate between isolates with respect to tissue tropism that could have given indications of differences in virulence. Hence, experimental challenge studies, using isolates with different VNTR profiles or MLST, have to be performed with respect to future knowledge of virulence factors and development of efficient vaccines.

## Conclusions

The present study tried to clarify aspects of transmission of *F. psychrophilum* in Chile and Norway using VNTRs as genetic markers, ie. a MLVA system*.* The method gave, to a large extent, the same resolution as that found in a previous MLST study Apablaza et al. [36], with a high diversity among Chilean isolates and less variation among the Norwegian isolates. The method was not able to separate isolates with respect to host species except for one cluster of distinct isolates from wild Atlantic salmon in Norway. It is not clear if this is due to limited resolution of the method or a result of high infection pressure between infected populations of Atlantic salmon and rainbow trout in Chile and Europe. The potential of the VNTR analysis as a method for separation of isolates with respect to virulence have to be tested in challenge experiments using isolates with different profiles. In addition, future studied should include more isolates of *F. psychrophilum* for a more detailed study of the resolution power of the two methods, MLST and VNTR analysis.

## Methods

### Isolates

#### Collection of *F. psychrophilum*

The isolates included in this study were obtained from different fish species, countries, and tissues mainly in the period from 2006 to 2012 (Table 1). A table with the information of the majority of isolates is presented in Apablaza et al. [36] and a complete overview of all the isolates is presented in Additional file 3. Fish referred to as trout (*Salmo trutta*) in this study include brown trout from rivers and sea trout.

This study was performed in strict accordance with the recommendations of the Norwegian Animal Welfare Act (01.01.2010) and the work followed the regulations set by The Norwegian Food Safety Authority. The bacterial samples were obtained from the Atlantic salmon after they had been anaesthetized by a blow to the head and killed by instantly decapitation. This procedure complies with Norwegian fish welfare regulations.

#### Isolation of *F. psychrophilum*

The *F. psychrophilum* isolates were cultured from different tissues using TYES-A added glucose (FLPA) [48]. The Chilean, the Danish and the Scottish isolates were transported in agar tubes to our laboratory. The Norwegian samples were cultured directly from fish tissues at the laboratory of the Fish Diseases Research Group of the University of Bergen. The procedures for culturing and identification of the isolates were explained in a previous work [36]. The agar plates were incubated at 15 °C for 48–72 h up to three weeks. After identification of the bacteria the isolates were preserved by adding glycerol 28 % and stored at −80 °C or in liquid nitrogen. DNA was extracted using a DNeasy kit (Qiagen, Hilden, Dusseldorf, Germany) as described by in the manufacturer’s instructions, and stored at -20 °C. To identify *F. psychrophilum* a set of specific primers for 16S rRNA gene were used [49]. PCR amplification, visualization, cleaning of PCR products, and sequencing were as described by Apablaza et al. 2013 [36].

The complete genome of *F. psychrophilum* JIP02/86 strain, obtained from GenBank (Accession number AM398681) was analysed for the presence of potential tandem repeat regions by the software Tandem Repeat Finder (Benson G, 1999). Loci with tandem repeats consisting of four to ten nucleotides were selected for the analysis. Specific oligonucleotide primers for 12 potential variable number tandem repeats (VNTR) loci were designed using the Vector NTI Suite 9.0 program package (InforMax Inc). The oligonucleotide primers had Tm ranging from 52 to 59° (Table 2).

The amplification of VNTRs was performed as described in Brevik et al. [26]. The PCR products were visualized using gel electrophoresis with 1 % agarose. In cases where the initial PCR did not amplify a target sequence, a second or third PCR was run. This included using previous PCR product as template in an identical PCR run (repeated PCR), or using a gradient of different Tm on extracted DNA to allow more specific binding of the primers. Additional pairs of flanking primers were designed for VNTR7-F/R and VNTR 13-F/R, since not all the isolates were amplified using the initial primers (Table 2). The cleaning and sequencing of PCR products were also performed as described in Brevik et al. [26]. Initially the 12 set of primers that identified potentially suitable VNTRs were tested using DNA extracted from 10 isolates from Norway and Chile and the type strain of *F. psychrophilum,* NCIMB 1947. Among these isolates eight VNTRs showed variation, while no variation was found in the latter four VNTRs. Bases on these results the remaining 42 isolates were tested for the eight former VNTRs only.

### VNTR stability

To test the stability in vitro of all the VNTRs at different temperatures the isolate No12-49-As-Op was tested after 12 passages and at incubation temperatures of 4 °C (No12-49-As-Op/4) and 15 °C (No12-49-As-Op/15).

### Phylogenetic analysis

The relationships between the type species and the *F. psychrophilum* isolates from Norway and Chile, based on the variation in the VNTRs, were analysed using neighbour-joining (NJ) distance method and principal component analysis (PCA).

At each VNTR locus in a single taxon, the VNTR was coded as a discrete character (i.e., 1–9, A-H) based on the specific number of repeats at the region in question. These allele profiles were used to construct a data matrix within the Mesquite System for Phylogenetic Analysis (Maddison, W.P. and D.R Maddison), [26].

### Principal component analysis

The principal component analysis (PCA) of VNTRs of all the isolates was performed in Unscrambler 9.8, CAMO, Oslo, Norway.

## Additional files

Additional file 1:
**Accesion numbers of all the VNTR sequences included in the study.**
Additional file 2:
**Allelic profile of the 53** ***F. psychrophilum*** **isolates included in this study.**
Additional file 3:
**Overview of the isolates of** ***F. psychrophilum*** **included in the study.**

## Abbreviations

BCWD: Bacterial cold water disease
DNA: Deoxyribonucleic acid
FLPA: TYES-A added glucose agar
MLST: Multi locus of sequence typing
MLVA: Multiple loci VNTR analysis
NJ: Neighbour-joining distance method
PCA: Principal component analysis
PCR: Polymerase chain reaction
PFGE: Pulsed-field electrophoresis
RTFS: Rainbow trout fry syndrome
VNTR: Variable number of tandem repeats
VS: VNTR set
