# Supplementary material for: Variable Number of Tandem Repeats (VNTR) analysis of Flavobacterium psychrophilum from salmonids in Chile and Norway
Source: BMC Vet Res. 2015 Jul 14;11:150. doi: 10.1186/s12917-015-0469-7 (PMC4501049; doi:10.1186/s12917-015-0469-7)
Supplement: Additional file 1: — Accesion numbers of all the VNTR sequences included in the study. [file 12917_2015_469_MOESM1_ESM.docx]

Overview of the isolates of *F. psychrophilum* included in the study

|  | Country | County | Date | Water source | Host | Wild/farmed | Sample |
| --- | --- | --- | --- | --- | --- | --- | --- |
| Ch06-1-Rt-G/F | Chile | Curarrehue | 2006 | river | rainbow trout | farmed | gills-fin |
| Ch07-2-As-G/W | Chile | Hornopiren | 2007 | estuary | Atlantic salmon | farmed | gills-wound |
| Ch07-3-As-G/F | Chile | Llanquihue | 2007 | lake | Atlantic salmon | farmed | gills-fin |
| Ch07-4-Rt-K/Sp | Chile | Rupanco | 2007 | lake | rainbow trout | farmed | k-s |
| Ch09-5-Rt-G/F | Chile | Valdivia | 2009 | river | rainbow trout | farmed | gills-fin |
| Ch09-6-As-K | Chile | Valdivia | 2009 | river | Atlantic salmon | farmed | kidney |
| Ch07-7-As-G/F | Chile | Llanquihue | 2007 | lake | Atlantic salmon | farmed | gills-fin |
| Ch08-8-As-G/F | Chile | Llanquihue | 2008 | lake | Atlantic salmon | farmed | gills-fin |
| Ch07-9-Rt-K | Chile | Antuco | 2007 | spring water | rainbow trout | farmed | kidney |
| Ch08-10-Rt-K | Chile | Melipeuco | 2008 | spring water | rainbow trout | farmed | kidney |
| Ch08-11-As-G/F | Chile | Puerto Cisnes | 2009 | estuary | Atlantic salmon | farmed | gills-fin |
| Ch09-12-Rt-W | Chile | Puerto Rosales | 2009 | lake | rainbow trout | farmed | wound |
| Ch09-13-As-W | Chile | Hornohuinco | 2009 | lake | Atlantic salmon | farmed | wound |
| Ch10-14-Rt-G | Chile | Panguipulli | 2010 | fresh water | rainbow trout | farmed | gill |
| Ch10-15-As-F | Chile | Chinquihue | 2010 | fresh water | Atlantic salmon | farmed | fin |
| Ch10-16-Rt-G | Chile | Quillaipe | 2010 | fresh water | rainbow trout | farmed | gill |
| Ch08-17-Rt-G/F | Chile | Talca | 2008 | river | rainbow trout | farmed | gills-fin |
| Ch10-18-Rt-G | Chile | Quillaipe | 2010 | fresh water | rainbow trout | farmed | gill |
| Ch08-19-As-G/F | Chile | Puerto Cisnes | 2008 | estuary | Atlantic salmon | farmed | gills-fin |
| Ch07-20-Rt-K | Chile | Quellon | 2007 | lake | rainbow trout | farmed | kidney |
| Ch10-21-Rt-W | Chile | LLanquihue | 2010 | lake | rainbow trout | farmed | wound |
| Ch10-22-As-nd | Chile | LLanquihue | 2010 | lake | Atlantic salmon | farmed | nd |
| Ch07-23-As-K/Sp | Chile | Rupanco | 2007 | lake | Atlantic salmon | farmed | kidney-spleen |
| Ch07-24-Rt-K | Chile | Antuco | 2007 | spring water | rainbow trout | farmed | kidney |
| Ch10-25-As-Sp | Chile | Hornopiren | 2010 | fresh water | Atlantic salmon | farmed | spleen |
| No09-26-As-K | Norway | Møre og Romsdal | 2009 | fresh water | Atlantic salmon | wild | kidney |
| No09-27-As-SK | Norway | Møre og Romsdal | 2009 | fresh water | Atlantic salmon | wild | skin |
| No09-28-Rt-K | Norway | Hordaland | 2009 | brackish water | rainbow trout | farmed | kidney |
| No09-29-As-nd | Norway | Hordaland | 2009 | fresh water | Atlantic salmon | wild | nd |
| No09-30-T-Sp | Norway | Rogaland | 2009 | fresh water | trout | wild | spleen |
| No09-31-As-Eg | Norway | Møre og Romsdal | 2009 | fresh water | Atlantic salmon | wild | eggs |
| No09-32-As-G | Norway | Møre og Romsdal | 2009 | fresh water | Atlantic salmon | wild | gills |
| No09-33-As-G | Norway | Møre og Romsdal | 2009 | fresh water | Atlantic salmon | wild | gills |
| No10-34-T-G | Norway | Møre og Romsdal | 2010 | fresh water | trout | wild | gills |
| No10-35-T-W | Norway | Møre og Romsdal | 2010 | fresh water | trout | wild | wound |
| No10-36-T-W | Norway | Møre og Romsdal | 2010 | fresh water | trout | wild | wound |
| No10-37-T-W | Norway | Møre og Romsdal | 2010 | fresh water | trout | wild | wound |
| No10-38-As-W | Norway | Hordaland | 2010 | fresh water | Atlantic salmon | farmed | wound |
| No10-39-As-W | Norway | Møre og Romsdal | 2010 | fresh water | Atlantic salmon | farmed | wound |
| No10-42-As-K | Norway | Hordaland | 2010 | fresh water | Atlantic salmon | wild | kidney |
| No10-43-As-K | Norway | Møre og Romsdal | 2010 | fresh water | Atlantic salmon | wild | kidney |
| No10-44-As-M | Norway | Hordaland | 2010 | fresh water | Atlantic salmon | wild | milt |
| No11-45-As-W | Norway | Hordaland | 2011 | sea water | Atlantic salmon | farmed | wound |
| No11-46-As-Sp | Norway | Hordaland | 2011 | fresh water | Atlantic salmon | wild | spleen |
| No12-49-As-Op | Norway | Nordland | 2012 | fresh water | Atlantic salmon | farmed | operculum |
| No12-50-As-Mo | Norway | Møre og Romsdal | 2012 | fresh water | Atlantic salmon | farmed | mouth |
| No12-51-As-W | Norway | Møre og Romsdal | 2012 | fresh water | Atlantic salmon | farmed | wound |
| Sc10-47-As-K | Scotland | NA | 2010 | fresh water/loch | Atlantic salmon | farmed | kidney |
| Sc11-48-Rt-Sp | Scotland | NA | 2011 | fresh water/burn | rainbow trout | farmed | spleen |
| Dn94-52-Rt-Sp | Denmark | Western Jutland | 1994 | fresh water | rainbow trout | farmed | spleen |
| Dn08-53-Rt-K | Denmark | Himmerland | 2008 | fresh water | rainbow trout | farmed | kidney |
| NCIMB 1947 | USA | Washington | NA | fresh water | coho salmon | NA | kidney |
| JIP02/86 | France | NA | 1986 | NA | rainbow trout | NA | kidney |

Isolates of *F. psychrophilum* from Norway, Chile, Scotland and Denmark collected between 1994 to 2012, from different water sources, fish species, farmed or wild fish and tissues. Codes indicate the country of isolation, Norway (No), Chile (Ch), Scotland (Sc), and Denmark (Dn); year (1994 to 2012), number of isolate (1 to 53), fish species, A. salmon (As), rainbow trout (Rt), trout (T) and tissue, gills (G), fins (F), wound (W), kidney (K), eggs (Eg), milt (M), spleen (Sp), skin (SK), operculum (Op). NA, no information is available. Nd, not determinated
