# Supplementary material for: Variable Number of Tandem Repeats (VNTR) analysis of Flavobacterium psychrophilum from salmonids in Chile and Norway
Source: BMC Vet Res. 2015 Jul 14;11:150. doi: 10.1186/s12917-015-0469-7 (PMC4501049; doi:10.1186/s12917-015-0469-7)
Supplement: Additional file 2: — Allelic profile of the 53 F. psychrophilum isolates included in this study. [file 12917_2015_469_MOESM2_ESM.docx]

Accesion numbers of all the VNTR sequences included in the study

| **Isolate** | **VNTR 1** | **VNTR 5** | **VNTR 6** | **VNTR 7** | **VNTR 8** | **VNTR 9** | **VNTR 10** | **VNTR 13** |
| --- | --- | --- | --- | --- | --- | --- | --- | --- |
| Ch06-1-Rt/G-F | KM998796 | KM871143 | KP121209 | KP121359 | KM871077 | KM672463 | KM603559 | KP209995 |
| Ch07-2-As/G-W | KM998785 | KM871134 | KP121185 | KP121357 | KM871066 | KM672452 | KM603548 | KP209984 |
| Ch07-3-As/G-F | KM998788 | KM871137 | KP121205 | KP121360 | KM871069 | KM672455 | KM603551 | KP209987 |
| Ch07-4-Rt/K-Sp | KM998790 | KM871121 | KP121167 | KP121381 | KM871071 | KM672457 | KM603553 | KP209989 |
| Ch09-5-Rt/G-F | KM998797 | KM871124 | KP121179 | KP121369 | KM871078 | KM672464 | KM603560 | KP209996 |
| Ch09-6-As/K | KM998798 | KM871144 | KP121194 | KP121361 | KM871079 | KM672465 | KM603561 | KP209997 |
| Ch07-7-As/G-F | KM998789 | KM871138 | KP121197 | KP121379 | KM871070 | KM672456 | KM603552 | KP209988 |
| Ch08-8-As/G-F | KM998795 | KM871142 | KP121193 | KP121365 | KM871076 | KM672462 | KM603558 | KP209994 |
| Ch07-9-Rt/K | KM998787 | KM871136 | KP121192 | KP121358 | KM871068 | KM672454 | KM603550 | KP209986 |
| Ch08-10-Rt/K | KM998799 | KM871145 | KP121198 | KP121363 | KM871080 | KM672466 | KM603562 | KP209998 |
| Ch08-11-As/G-F | KM998794 | KM871141 | KP121196 | KP121372 | KM871075 | KM672461 | KM603557 | KP209993 |
| Ch09-12-Rt-W | KM998807 | KM871148 | KP121190 | KP121374 | KM871088 | KM672474 | KM603570 | KP210004 |
| Ch09-13-As-W | KM998784 | KM871120 | KP121161 | KP121367 | KM871065 | KM672451 | KM603547 | KP209983 |
| Ch10-14-Rt-G | KM998802 | KM871130 | KP121202 | KP121368 | KM871083 | KM672469 | KM603565 | KP210000 |
| Ch10-15-As-F | KM998803 | KM871123 | KP121180 | KP121349 | KM871084 | KM672470 | KM603566 | KP210023 |
| Ch10-16-Rt-G | KM998801 | KM871172 | KP121201 | KP121383 | KM871082 | KM672468 | KM603564 | KP209999 |
| Ch08-17-Rt/G-F | KM998800 | KM871122 | KP121183 | KP121348 | KM871081 | KM672467 | KM603563 | KP210022 |
| Ch10-18-Rt-G | KM998804 | KM871131 | KP121200 | KP121387 | KM871085 | KM672471 | KM603567 | KP210001 |
| Ch08-19-As/G-F | KM998792 | KM871139 | KP121207 | KP121378 | KM871073 | KM672459 | KM603555 | KP209991 |
| Ch07-20-Rt/K | KM998793 | KM871140 | KP121208 | KP121364 | KM871074 | KM672460 | KM603556 | KP209992 |
| Ch10-21-Rt-W | KM998805 | KM871146 | KP121195 | KP121362 | KM871086 | KM672472 | KM603568 | KP210002 |
| Ch10-22-As-nd | KM998806 | KM871147 | KP121203 | KP121366 | KM871087 | KM672473 | KM603569 | KP210003 |
| Ch07-23-As/K-Sp | KM998791 | KM871171 | KP121206 | KP121382 | KM871072 | KM672458 | KM603554 | KP209990 |
| Ch07-24-Rt/K | KM998786 | KM871135 | KP288498 | KP121356 | KM871067 | KM672453 | KM603549 | KP209985 |
| Ch10-25-As-Sp | KP288497 | KM871119 | KP121159 | KP121341 | KM871064 | KM672450 | KM603546 | KP209982 |
| No09-26-As-K | KM998818 | KM871157 | KP121182 | KP121389 | KM871103 | KM672489 | KM603585 | KP210009 |
| No09-27-As-SK | KP288494 | KM871129 | KP288499 | KP121391 | KM871100 | KM672486 | KM603582 | KP210020 |
| No09-28-Rt-K | KM998824 | KM871161 | KP121210 | KP121384 | KM871109 | KM672495 | KM603591 | KP210012 |
| No09-29-As-nd | KM998811 | KM871152 | KP121171 | KP121342 | KM871092 | KM672478 | KM603574 | KP210030 |
| No09-30-T-Sp | KM998819 | KM871132 | KP121165 | KP121355 | KM871104 | KM672490 | KM603586 | KP210035 |
| No09-31-As-Eg | KM998814 | KM871153 | KP121186 | KP121380 | KM871097 | KM672483 | KM603579 | KP210007 |
| No09-32-As-G | KM998815 | KM871154 | KP121184 | KP121388 | KM871098 | KM672484 | KM603580 | KP210018 |
| No09-33-As-G | KM998816 | KM871128 | KP121163 | KP121390 | KM871099 | KM672485 | KM603581 | KP210019 |
| No10-34-T-G | KM998812 | KM871173 | KP121160 | KP121393 | KM871093 | KM672479 | KM603575 | KP210024 |
| No10-35-T-W | KM998813 | KM871125 | KP121162 | KP288501 | KM871094 | KM672480 | KM603576 | KP210015 |
| No10-36-T-W | KM998834 | KM871126 | KP121164 | KP121394 | KM871095 | KM672481 | KM603577 | KP210017 |
| No10-37-T-W | KP288496 | KM871127 | KP121166 | KP121371 | KM871096 | KM672482 | KM603578 | KP210016 |
| No10-38-As-W | KM998820 | KM871158 | KP121174 | KP121354 | KM871105 | KM672491 | KM603587 | KP210036 |
| No10-39-As-W | KM998831 | KM871168 | KP121170 | KP121344 | KM871116 | KM672502 | KM603598 | KP210026 |
| No10-42-As-K | KM998808 | KM871149 | KP121168 | KP121350 | KM871089 | KM672475 | KM603571 | KP210029 |
| No10-43-As-K | KM998817 | KM871155 | KP121181 | KP121370 | KM871101 | KM672487 | KM603583 | KP210008 |
| No10-44-As-M | KM998832 | KM871169 | KP121176 | KP121352 | KM871117 | KM672503 | KM603599 | KP210027 |
| No11-45-As-W | KM998822 | KM871160 | KP121189 | KP121377 | KM871107 | KM672493 | KM603589 | KP210011 |
| No11-46-As-Sp | KM998833 | KM871170 | KP121177 | KP121353 | KM871118 | KM672504 | KM603600 | KP210028 |
| No12-49-As-Op-15C | KM998825 | KM871162 | KP121175 | KP121345 | KM871110 | KM672496 | KM603592 | KP210032 |
| No12-49-As-Op-4C | KM998826 | KM871163 | KP121172 | KP121346 | KM871111 | KM672497 | KM603593 | KP210033 |
| No12-49-As-Op | KM998827 | KM871164 | KP121173 | KP121347 | KM871112 | KM672498 | KM603594 | KP210034 |
| No12-50-As-Mo | KM998830 | KM871167 | KP121169 | KP121343 | KM871115 | KM672501 | KM603597 | KP210025 |
| No12-51-As-W | KP288495 | KM871156 | KP288500 | KP121351 | KM871102 | KM672488 | KM603584 | KP210031 |
| Sc10-47-As-K | KM998828 | KM871165 | KP121199 | KP121385 | KM871113 | KM672499 | KM603595 | KP210013 |
| Sc11-48-Rt-Sp | KM998829 | KM871166 | KP121204 | KP121386 | KM871114 | KM672500 | KM603595 | KP210014 |
| Dn94-52-Rt-Sp | KM998810 | KM871150 | KP121191 | KP121373 | KM871091 | KM672477 | KM603573 | KP210006 |
| Dn08-53-Rt-K | KM998809 | KM871151 | KP121187 | KP121376 | KM871090 | KM672476 | KM603572 | KP210005 |
| NMCB1947 | KM998823 | KM871133 | KP121178 | KP121392 | KM871108 | KM672494 | KM603590 | KP210021 |
| JIP02/86 | KM998821 | KM871159 | KP121188 | KP121375 | KM871106 | KM672492 | KM603588 | KP210010 |
