# Supplementary material for: Variable Number of Tandem Repeats (VNTR) analysis of Flavobacterium psychrophilum from salmonids in Chile and Norway
Source: BMC Vet Res. 2015 Jul 14;11:150. doi: 10.1186/s12917-015-0469-7 (PMC4501049; doi:10.1186/s12917-015-0469-7)
Supplement: Additional file 3: — Overview of the isolates of F. psychrophilum included in the study. [file 12917_2015_469_MOESM3_ESM.docx]

Allelic profile of the 53 *F. psychrophilum* isolates included in this study

| Isolate | VNTR-1 | VNTR-5 | VNTR-6 | VNTR-7 | VNTR-8 | VNTR-9 | VNTR-10 | VNTR-13 | VS |
| --- | --- | --- | --- | --- | --- | --- | --- | --- | --- |
| Ch07-2-As/G-W | 4r | 5r | 2r | 3r | 6r | 7r | 2r | 3r | 1 |
| Ch09-6-As/K | 4r | 5r | 2r | 3r | 6r | 7r | 2r | 3r | 1 |
| Ch08-8-As/G-F | 4r | 5r | 2r | 3r | 6r | 7r | 2r | 3r | 1 |
| Ch08-11-As/G-F | 4r | 5r | 2r | 3r | 6r | 7r | 2r | 3r | 1 |
| Ch08-19-As/G-F | 4r | 5r | 2r | 3r | 6r | 7r | 2r | 3r | 1 |
| Ch07-20-Rt/K | 4r | 5r | 2r | 3r | 6r | 7r | 2r | 3r | 1 |
| Sc10-47-As-K | 4r | 5r | 2r | 3r | 6r | 7r | 2r | 3r | 1 |
| No09-31-As-Eg | 4r | 5r | 2r | 3r | 6r | 3r | 2r | 3r | 2 |
| No10-43-As-K | 4r | 5r | 2r | 3r | 6r | 3r | 2r | 3r | 2 |
| Ch08-10-Rt/K | 4r | 5r | 2r | 3r | 5r | 7r | 2r | 3r | 3 |
| Ch09-12-Rt-W | 4r | 5r | 2r | 3r | 5r | 7r | 2r | 3r | 3 |
| No09-28-Rt-K | 4r | 5r | 2r | 3r | 5r | 7r | 2r | 3r | 3 |
| No11-45-As-W | 4r | 5r | 2r | 3r | 5r | 7r | 2r | 3r | 3 |
| Sc11-48-Rt-Sp | 4r | 5r | 2r | 3r | 5r | 7r | 2r | 3r | 3 |
| Dn94-52-Rt-Sp | 4r | 5r | 2r | 3r | 5r | 7r | 2r | 3r | 3 |
| No10-38-As-W | 4r | 5r | 2r | 2r | 4r | 3r | 2r | 0r | 4 |
| No10-42-As-K | 4r | 5r | 2r | 2r | 4r | 3r | 2r | 0r | 4 |
| Ch10-16-Rt-G | 3r | 6r | 2r | 3r | 5r | 7r | 2r | 3r | 5 |
| Ch07-23-As/K-Sp | 3r | 6r | 2r | 3r | 5r | 7r | 2r | 3r | 5 |
| Ch06-1-Rt/G-F | 3r | 5r | 2r | 3r | 5r | 6r | 2r | 3r | 6 |
| Ch07-3-As/G-F | 3r | 5r | 2r | 3r | 5r | 6r | 2r | 3r | 6 |
| Ch07-7-As/G-F | 3r | 5r | 2r | 3r | 5r | 6r | 2r | 3r | 6 |
| Ch07-9-Rt/K | 3r | 5r | 2r | 3r | 5r | 6r | 2r | 3r | 6 |
| Ch07-24-Rt/K | 3r | 5r | 2r | 3r | 5r | 6r | 2r | 3r | 6 |
| Ch10-21-Rt-W | 3r | 5r | 2r | 3r | 7r | 6r | 2r | 3r | 7 |
| Ch10-22-As-nd | 3r | 5r | 2r | 3r | 7r | 6r | 2r | 3r | 7 |
| No09-29-As-nd | 3r | 5r | 2r | 2r | 4r | 3r | 2r | 0r | 8 |
| No10-39-As-W | 3r | 5r | 2r | 2r | 4r | 3r | 2r | 0r | 8 |
| No10-44-As-M | 3r | 5r | 2r | 2r | 4r | 3r | 2r | 0r | 8 |
| No11-46-As-Sp | 3r | 5r | 2r | 2r | 4r | 3r | 2r | 0r | 8 |
| No12-50-As-Mo | 3r | 5r | 2r | 2r | 4r | 3r | 2r | 0r | 8 |
| No12-51-As-W | 3r | 5r | 2r | 2r | 4r | 3r | 2r | 0r | 8 |
| No12-49-As-Op | 3r | 5r | 2r | 2r | 4r | 3r | 2r | 0r | 8 |
| No12-49-As-Op/4C | 3r | 5r | 2r | 2r | 4r | 3r | 2r | 0r | 8 |
| No12-49-As-Op/15C | 3r | 5r | 2r | 2r | 4r | 3r | 2r | 0r | 8 |
| No09-27-As-SK | 3r | 3r | 2r | 0r | 4r | 3r | 3r | 2r | 9 |
| No09-33-As-G | 3r | 3r | 2r | 0r | 4r | 3r | 3r | 2r | 9 |
| No10-36-T-W | 3r | 3r | 2r | 0r | 4r | 3r | 3r | 2r | 9 |
| Ch10-15-As-F | 3r | 1r | 2r | 1r | 6r | 3r | 2r | 1r | 10 |
| Ch08-17-Rt/G-F | 3r | 1r | 2r | 1r | 6r | 3r | 2r | 1r | 10 |
| Ch07-4-Rt/K-Sp | 5r | 1r | 2r | 3r | 6r | 3r | 2r | 3r | 11 |
| Dn08-53-Rt-K | 4r | 5r | 2r | 3r | 5r | 9r | 2r | 3r | 12 |
| No09-32-As-G | 4r | 5r | 2r | 0r | 6r | 3r | 2r | 2r | 13 |
| No09-26-As-K | 4r | 5r | 2r | 0r | 6r | 3r | 2r | 3r | 14 |
| jip02/86 | 4r | 5r | 2r | 3r | 8r | 7r | 2r | 3r | 15 |
| Ch09-13-As-W | 4r | 4r | 2r | 3r | 5r | 3r | 2r | 3r | 16 |
| Ch10-18-Rt-G | 4r | 4r | 2r | 0r | 6r | 3r | 2r | 3r | 17 |
| Ch10-14-Rt-G | 4r | 4r | 2r | 3r | 6r | 3r | 2r | 3r | 18 |
| No10-34-T-G | 3r | 6r | 2r | 0r | 5r | 3r | 2r | 0r | 19 |
| No09-30-T-Sp | 3r | 4r | 2r | 4r | 6r | 3r | 2r | 0r | 20 |
| NCIMB 1947 | 3r | 4r | 2r | 0r | 9r | 3r | 2r | 2r | 21 |
| No10-35-T-W | 3r | 3r | 2r | 1r | 4r | 3r | 3r | 2r | 22 |
| No10-37-T-W | 3r | 3r | 2r | 3r | 4r | 3r | 3r | 2r | 23 |
| Ch09-5-Rt/G-F | 3r | 2r | 3r | 3r | 8r | 3r | 2r | 3r | 24 |
| Ch10-25-As-Sp | 3r | 1r | 2r | 2r | 6r | 3r | 2r | 3r | 25 |

Allelic profiles are presented as number of repeats (r). Identical VT profiles are numbered from 1 to 10. Unique alleles are numbered from 11 to 25. VNTR-1, VNTR-5 and VNTR-7 have a deletion in two nucleotides in one repeat (=0.7), AGGCAAT to AGGCA; ATTAGGC to ATTAG; and AAAAATT to AAAAA, respectively. VNTR- 9 has a deletion of one nucleotide in one repeat (TTTG to TTT=0.8), and VNTR-10 has deletions in three nucleotides (CTTCTTTTA to CTT=0.3).The allelic profile includes the isolate analysed for VNTR stability (No12-49-As-Op in 4°C and 15°C).
